# Necrosis Is the Dominant Cell Death Pathway in Uropathogenic *Escherichia coli* Elicited Epididymo-Orchitis and Is Responsible for Damage of Rat Testis

**DOI:** 10.1371/journal.pone.0052919

**Published:** 2013-01-02

**Authors:** Yongning Lu, Sudhanshu Bhushan, Svetlin Tchatalbachev, Marcelo Marconi, Martin Bergmann, Wolfgang Weidner, Trinad Chakraborty, Andreas Meinhardt

**Affiliations:** 1 Department of Anatomy and Cell Biology, Unit of Reproductive Biology, Justus-Liebig-University Giessen, Giessen, Germany; 2 Department of Medical Microbiology, Justus-Liebig-University Giessen, Giessen, Germany; 3 Clinic and Policlinic of Urology, Pediatric Urology and Andrology, Justus-Liebig-University Giessen, Giessen, Germany; 4 Institute of Veterinary Anatomy, Histology, and Embryology, Justus-Liebig-University Giessen, Giessen, Germany; University Hospital of Münster, Germany

## Abstract

Male infertility is a frequent medical condition, compromising approximately one in twenty men, with infections of the reproductive tract constituting a major etiological factor. Bacterial epididymo-orchitis results in acute inflammation most often caused by ascending canalicular infections from the urethra via the continuous male excurrent ductal system. Uropathogenic *Escherichia coli* (UPEC) represent a relevant pathogen in urogenital tract infections. To explore how bacteria can cause damage and cell loss and thus impair fertility, an *in vivo* epididymo-orchitis model was employed in rats by injecting UPEC strain CFT073 into the vas deference in close proximity to the epididymis. Seven days post infection bacteria were found predominantly in the testicular interstitial space. UPEC infection resulted in severe impairment of spermatogenesis by germ cell loss, damage of testicular somatic cells, a decrease in sperm numbers and a significant increase in TUNEL (+) cells. Activation of caspase-8 (extrinsic apoptotic pathway), caspase-3/−6 (intrinsic apoptotic pathway), caspase-1 (pyroptosis pathway) and the presence of 180 bp DNA fragments, all of which serve as indicators of the classical apoptotic pathway, were not observed in infected testis. Notably, electron microscopical examination revealed degenerative features of Sertoli cells (SC) in UPEC infected testis. Furthermore, the passive release of high mobility group protein B1 (HMGB1), as an indication of necrosis, was observed *in vivo* in infected testis. Thus, necrosis appears to be the dominant cell death pathway in UPEC infected testis. Substantial necrotic changes seen in Sertoli cells will contribute to impaired spermatogenesis by loss of function in supporting the dependent germ cells.

## Introduction

The mammalian testis is essentially composed of two main compartments, ie. the interstitial space with the androgen-producing Leydig cells and leukocytes and the seminiferous tubules containing the developing germ cells in close physical association with the columnar Sertoli cells. In the interstitial space, testicular macrophages act as a first line of defense [1], [2], whilst in the seminiferous epithelium the Sertoli cells, beside their role in supporting spermatogenesis, are undoubtedly of considerable importance in the control of immune response against pathogens arising from the ductal system. The recent discovery of microbial pattern recognition receptors such as Toll-like receptor (TLR) on Sertoli cells together with their ability to produce inflammatory mediators, places them in a central position to orchestrate protection from ascending canalicular microbial infection [3]–[5]. In turn many of the negative effects of infection/inflammation on spermatogenesis may be attributed to impaired Sertoli cell function with subsequent disruptive effects on germ cell development and survival [6], [7].

Given the predominant occurrence of uropathogenic *E. coli* (UPEC) with urinary tract infections, it is not surprising that *E. coli* (apart from other sexually transmitted microbes) is the most frequently isolated pathogen from urine and semen samples of patients with prostatitis and epididymo-orchitis [8]–[10]. Direct characterization and analysis of bacterial traits or virulence genes such as alpha-hemolysin (HlyA) confirmed the relevance of uropathogenic *E. coli* (UPEC) in infectious male infertility and subfertility which overall ranks first amongst the known reasons for male factor infertility preceded only by idiopathic causes [11]–[13].

In men, bacterial epididymo-orchitis is treated with antibiotic and antiphlogistic pharmacotherapy. Of note, even after eradication of the pathogen by antibiotic treatment, about 50% of men do not recover normal sperm counts. As animal experiments indicate an underlying reason could be the silent continuation of inflammation that can affect both testes causing permanent impairment of fertility by germ cell loss or alternatively duct obstruction [14].

Cell loss following microbial infection is often the consequence of programmed cell death (apoptosis and pyroptosis). Apoptosis is mainly mediated through one of two signaling cascades termed the intrinsic and the extrinsic pathway. The extrinsic pathway is initiated by the binding of death receptors to their cognate ligands leading to the recruitment of FAS-associated death domain protein (FADD) and pro-caspase 8, followed by dimerization and activation of caspase 8, which then directly cleave and activate executor caspases 3 and 7. Alternatively, the intrinsic pathway is activated by stimuli that lead to outer mitochondrial membrane permeabilization and activation of procaspase-9. Active caspase-9 then in turn activates the executioner caspases-3, -6 and -7. Bacteria are able to trigger apoptosis by a variety of mechanism that include virulence factors (e.g. S*taphylococcus aureus*, *Listeria monocytogenes* ) or by repressing critical host survival pathways (*Yersinia enterocolica*, *Salmonella typhimurium*) [15]. UPEC were shown to induce apoptotic pathways by secretion of hemolysins as virulence factor resulting in suppression of NF-κB activation, decreased secretion of proinflammatory cytokines and recruitment of neutrophils [16]. On the other hand, UPEC were able to induce cell death of renal tubular cells in a caspase-independent manner [17]. Unlike the formation of membrane bounded apoptotic bodies observed during apoptosis, plasma membrane integrity is rapidly compromised in necrotic cells and results in spilling of intracellular contents into extracellular space. Therefore, necrosis inevitably affects neighboring cells, usually provoking significant inflammatory response and causing tissue injury. It is increasingly recognized that apoptosis and necrosis can coexist in the same tissue or even in the same cell type and that high-mobility group box-1 protein (HMGB1) is a useful biochemical indicator of necrosis, including pathogen induced necrosis [18]. Under physiological conditions, HMGB1 is a chromatin binding nuclear protein that remains firmly attached to chromatin in apoptotic cells even after undergoing secondary necrosis and partial autolysis [19]. However, in necrotic cells HMGB1 is passively released into the cytoplasm and subsequently extracellular space, where it serves as a late proinflammatory molecule [19].

Infertility affects approximately one in six couples worldwide with roughly half of the cases being attributed to a male factor. In view of the importance of infection in the etiology of male fertility disturbances it is surprising that relatively little is known how pathogens cause damage. Thus it was the aim of this study to elucidate mechanism how bacteria can impair testicular function.

## Materials and Methods

### Animals

Adult male Wistar rats (249∼270 g) were purchased from Harlan (Borchen, Germany) and kept at 22°C with 12 h light: 12 h dark schedule and fed with standard food pellets and water *ad libitum*. This study was carried out in strict accordance with the recommendations in the Guide for the Care and Use of Laboratory Animals of the German law of animal welfare. The protocol was approved by the Committee on the Ethics of Animal Experiments of the Regierungspraesidium Giessen, Giessen, Germany (permit number GI 20/23–No. 16/2009).

### Propagation of Bacteria

Uropathogenic *E. coli* strain CFT 073 was propagated overnight on Columbia blood agar plates (Oxoid, Wesel, Germany). Fresh cultures were inoculated in LB medium and grown to early exponential phase (OD600 = 0.4∼0.8) at 37°C in a shaker incubator. The concentration of viable bacteria was calculated using growth curves. Bacteria (2×10^9^ cfu) were centrifuged at 4,500×g for 8 min at room temperature. The pellet was washed once at room temperature with PBS and diluted again in 10 ml PBS.

### Bacterial Induced Experimental Epididymo-orchitis

Bacterial epididymo-orchitis was elicited in male Wistar rats as previously described [20]. Briefly, after general anesthesia, a scrotal incision was made to expose the testis, epididymis and vas deferens. Hundred µl of UPEC CFT073-saline suspension (about 4×10^6^ bacteria) was injected bilaterally into the vas deferens proximal to the cauda epididymis using 30-gauge needles. Sham operated rats were injected with saline. The vasa deferentia were ligated at the site of injection to prevent spreading of infection. After operation, animals were kept in standard housing condition until being sacrificed with an overdose of isoflurane in the morning of day 7 post injection. Both testicles and epididymides were removed aseptically with weight and volume determined.

### Determination of Testicular Infection

The testes from saline injected sham control and UPEC infected rats were homogenized in 10 ml sterile PBS using a sterile glass potter. Testicular homogenate (100 µl) from each sample were streaked on agar plate and incubated overnight at 37°C. Bacterial colonies were counted the next morning.

### Histological Evaluation

For histopathological assessment, sections of Bouins fixed and paraffin embedded testis and epididymis were stained with hematoxylin and eosin. Two sections from different parts of each testis were used for histopathological examination. Integrity of spermatogenesis was evaluated in 25∼30 randomly selected seminiferous tubules from each section.

### TUNEL Assay

DNA fragmentation of the testis was assessed semi-quantitatively with terminal deoxynucleotidyl transferase dUTP nick end labeling (TUNEL) assay using the ApopTag® Fluorescein *In Situ* Apoptosis Detection Kit (Millipore, MA, USA) following the manufacture’s instruction. Anti-digoxigenin conjugated antibody was used to visualize cells with DNA breakage. Labeled cryosections were finally examined using fluorescence microscopy (Axioplan 2 Imaging system, Carl Zeiss, Göttingen, Germany) using a 512∼542 nm filter.

### DNA Isolation

For PCR analysis of the UPEC specific pili gene PapC, total DNA was isolated from the testes with QIAamp® DNA Mini kit (Qiagen, Hilden, Germany; for PCR conditions and primer sequence see Table 1). Briefly, 1 µl of DNA sample (200 ng/µl) from each sample was used for PCR reaction. The PCR conditions were as follows: an initial denaturation step at 95°C for 5 min followed by 35 cycles of denaturation at 95°C for 30 s, annealing at 62°C for 30 s, extension at 72°C for 1 min, followed by a extension at 72°C for 10 min. The PCR product was electrophoresed on 1.5% agarose gel.

**Table 1:** Primer information.

Gene	Primer Sequence	Annealing Temperature	Accession No.	Amplicon Size (bp)
BCL-2	FP 5′-GGGATGCCTTTGTGGAACTA-3′ RP 5′-CTCACTTGTGGCCCAGGTAT-3′	59.6°C	NM_016993	138
BID	FP 5′-CGACGAGGTGAAGACATCCT-3′ RP 5′-AGACGTCACGGAGCAGAGAT-3′	59.6°C	NM_022684	119
BAX	FP 5′-TGTTTGCTGATGGCAACTTC-3′ RP 5′-GATCAGCTCGGGCACTTTAG-3′	59.6°C	NM_017059	104
BIM	FP 5′-AGATACGGATCGCACAGGAG-3′ RP 5′-ACCAGACGGAAGATGAATCG-3′	59.6°C	NM_171989	148
BAK1	FP 5′-GGGAAGACCCTCACCTTCTC-3′ RP 5′-ACATTGCAACCAGATCCACA-3′	59.6°C	NM_053812	142
UPEC PapC	FP 5′-GACGGCTGTACTGCAGGGTGTGGCG-3′ RP 5′-ATATCCTTTCTGCAGGGATGCAATA-3′	62.0°C	NC_04431	328
IL-1α	FP 5′-CCGGGTGGTGGTGTCAGCAA-3′ RP:5′-GCTGTGAGGTGCTGATCTGGGT-3′	61.8°C	NM_017019	148
IL-1ß	FP:5′-TGCCTCGTGCTGTCTGACCCA-3′ RP:5′-AGGCCCAAGGCCACAGGGAT-3′	61.8°C	NM_031512	137
TNF-α	FP:5′-GCCTCTTCTCATTCCTGCTC-3′ RP:5′-CCCATTTGGGAACTTCTCCT-3′	59.6°C	NM_012675	101
IL-6	FP:5′-TCCTACCCCAACTTCCAATGCTC-3′ RP:5′-TTGGATGGTCTTGGTCCTTAGCC-3′	59.6°C	NM_012589	79
ß2 macroglobulin	FP 5′-CCGTGATCTTTCTGGTGCTT-3′ RP 5′-AAGTTGGGCTTCCCATTCTC-3′	60.0°C	NM_012512	109

For the detection of DNA degradation pattern about 20 mg tissue from each testis sample were lysed in 600 µl lysis buffer (50 mM Tris-HCl; 400 mM NaCl; 100 mM EDTA; 0.5% SDS; 0.5 mg/ml proteinase K, pH 8.0). Proteins were removed by adding 200 µl of 5 M NaCl and subsequent centrifugation. DNA was precipitated and dissolved in 1×TE buffer (10 mM Tris-HCl, 1 mM EDTA, pH 8.0), followed by RNase A treatment at 37°C for 1 h. After re-precipitation and resuspension equal amounts of DNA samples were electrophoretically separated on 1.5% agarose gels and stained with ethidium bromide. A positive control for apoptosis-induced DNA laddering was generated by treating RAW 264.7 macrophages with 0.5 mM H_2_O_2_ for 24 h. Random DNA degradation resulting in “DNA smear” on agarose gels characteristic of necrotic cells was generated by repeatedly freeze – thawing RAW 264.7 macrophages.

### Quantitative Real Time PCR

Total RNA was extracted from UPEC infected testis by using RNeasy mini kit (Qiagen). Two µg RNA was reverse transcribed using oligo-dT primer and Moloney murine leukemia virus reverse transcriptase (M-MLV RT) in 40 µl volume. Quantitative real time PCR amplifications were performed by using an iCycler iQ® system (Bio-Rad, Munich, Germany) with iQ™ SYBR® Green supermix (Bio-Rad, Munich, Germany). PCR products were examined on agarose gels for specific amplification. The relative quantification of PCR products was determined by the comparative Ct method. The mRNA expression of all investigated genes was normalized by the non-regulated reference gene ß-2 microglobulin (ß2M). B2M expression was unaffected by treatments and correlated with the amount of RNA used for reverse transcription. Data were presented as relative expression (RE): RE = 2^ΔCt^, ΔCt = Ct_target gene_-Ct_ß2M_.

### Immunoblotting

For Western blot analysis 100 mg of testis was homogenized in RIPA buffer (25 mM Tris-HCl pH 7.6, 150 mM NaCl, 1% NP-40, 1% Sodium deoxycholate and 0.1% SDS) supplemented with proteinase inhibitor cocktail (Sigma Aldrich, Steinheim, Germany). Thirty µg of protein was resolved by 15% SDS-PAGE with subsequent blotting on nitrocellulose membranes (Hybond™ ECL™ 0.2 µm, GE Healthcare, UK). Blots were probed with primary antibodies in 5% nonfat milk overnight at 4°C. Immunoreactive proteins were detected with horseradish peroxidase-conjugated anti-rabbit or anti-mouse IgG using enhanced chemiluminescence (GE Healthcare). Membranes were stripped and re-probed with mouse anti-β-actin antibody (Sigma-Aldrich, Steinheim, Germany) as loading control. Rabbit anti-caspase-1 (Santa Cruz, CA), anti-caspase-3 (Abcam, Cambridge, UK), anti-caspase-6, anti-caspase-8, mouse anti-IkBα (all from Cell Signaling Technology, MA) and anti-HMGB1 (Abcam) were used as primary antibodies.

### Immunofluorescence Staining

Frozen tissue sections (10 µm) were fixed with ice cold methanol and then permeabilized with 0.2% Triton-X 100. Tissue sections were incubated with blocking agent (5% BSA +5% normal horse/sheep serum) for 1 h at room temperature, followed by incubation with primary antibodies at 4°C overnight. Rinsed samples were incubated with anti-rabbit or anti-mouse Cy-3 conjugated secondary antibody (1∶1000 dilution) for 1 h at room temperature in the dark and mounted with Vectashield ® mounting medium containing DAPI (Vector Laboratories, CA). Rabbit anti-*E. coli* antibody (Abcam), rabbit anti-HMGB1 antibody and mouse anti-p65 antibody (Santa Cruz) were used as primary antibodies.

### Electron Microscopy

Anesthetized animals were perfused via the left ventricle with either 2% glutaraldehyde and 2% formaldehyde in 0.1 mol/l sodium cacodylate buffer (pH 7.3) as fixative. To investigate the integrity of the blood-testis barrier and blood-epididymis barrier 1% lanthanum nitrate as established tracer was added to the fixative. Testicular and epididymal specimens (1 mm^3^) were excised and put in fixative for 1 h and subsequently in 1% osmium tetroxide for another 3 h. Tissue blocks were embedded in Epon 812 following dehydration. Semithin sections (1 µm) were stained with toluidine blue, ultrathin sections (60 nm) were contrasted using uranyl acetate and lead citrate for subsequent electron microscopical examination.

## Results

### UPEC Invasion and Localization in the Testis

**Figure 1:**
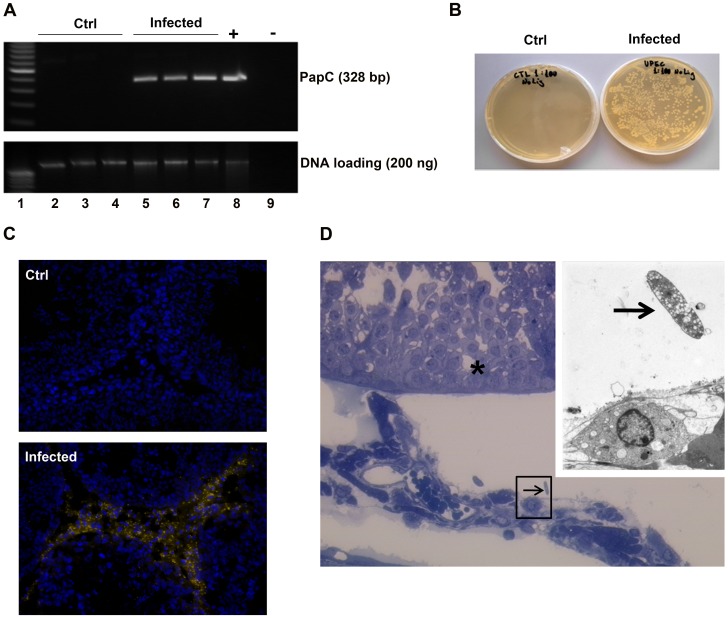
Presence of UPEC inside testes of infected rats 7 days after inoculation in the vas deferens. (**A**) Genomic DNA was extracted from testicular tissue and 200 ng DNA from each sample were amplified with PCR using UPEC pili primers. DNA isolated from one explanted testis immediately after direct UPEC injection served as a positive control. PCR products were separated on 1.5% agarose gel and stained with ethidium bromide. The same amount of DNA from each sample without PCR amplification was subjected to agarose gel electrophoresis and served as a loading control. Lane 1∶100 bp DNA marker; lane 2–4: samples from saline injected animals; Lane 5∼7: samples from UPEC infected rat testes; Lane 8: UPEC positive control; Lane 9: negative control. (**B**) Testicular homogenates from saline injected (left panel) and UPEC infected rats (right panel) were streaked on agar plates without antibiotics and kept at 37°C overnight. Colonies were counted under translucent light. (**C**) Cryosections of testis from control (left panel) and UPEC infected rats (right panel) were probed with anti-*E. coli* antibody and decorated with secondary anti-rabbit IgG antibody conjugated to Cy-3 (orange). DAPI (blue) was used for nuclear counterstain (x20 objective). (**D**) Semithin cross-section of a seminiferous tubule (asterisk) with adjacent interstitial space. Microbial presence in interstitial space is visible (arrow in the black frame, x20 objective). Inset: Electron microscopical examination on the same area (primary magnification x3,000).

To analyze the presence and localization of UPEC in the testis a number of independent techniques were applied. On the DNA level, the presence of the UPEC pili gene encoded by *PapC* revealed an amplicon of the expected size of 328 bp in DNA isolated from infected animals, but not in samples of sham operated controls (Figure 1A).

Furthermore, streaking testicular homogenates on agar plates followed by overnight incubation generated numerous yellowish-white bacterial colonies exclusively in infected testes samples (Figure 1B). Interestingly, using fluorescence microscopy bacteria labeled with anti-*E. coli* antibody were mainly observed in the testicular interstitial compartment with rare occurrence inside the lumen of the seminiferous tubules (Figure 1C). In agreement, electron microscopical examination confirmed the presence of *E. coli* mainly within the interstitial space of infected testis (Figure 1D).

### Integrity of BTB and BEB in UPEC Infected Rats

**Figure 2:**
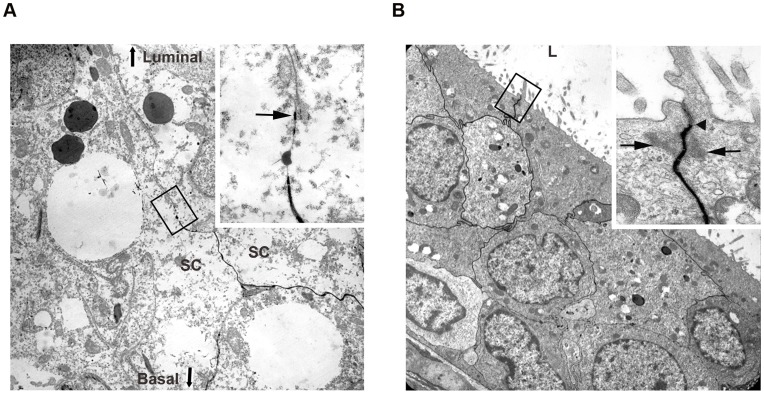
Electron microscopical analysis reveals intact blood-testis barrier (BTB) and blood-epididymis barrier (BEB). (**A**) Ultrastructural analysis shows that intercellular tracer penetration does not extend beyond the junctional complex of the BTB (arrow in inset) within the seminiferous epithelium of UPEC infected rats (x3,000 magnification, inset x20,000 magnification). SC = Sertoli cells, orientation of the luminal and basal compartment are highlighted (**B**) Ultrastructural analysis of a UPEC infected epididymis demonstrates intercellular tracer penetration (x3,000 magnification). Inset is a magnification of the area represented in the black frame showing the tight junctions. (x20,000 magnification).

The results above suggest that UPEC are able to migrate from the lumen across the seminiferous tubule wall to reach the testicular interstitial space within 7 days post infection of the vas deferens. To explore whether a disruption of the junctions forming the blood-testis (BTB) and blood-epididymis barrier (BEB) may facilitate passage of UPEC through the respective epithelial layers, electron microscopy of tracer (lanthanum) perfused infected testes (Figure 2A) and epididymides (Figure 2B) was performed. At 7 days post-infection, no breach of the BTB or BEB was observed as the lanthanum tracer did not pass beyond the tight junctions of the respective junctions following perfusion. These results suggest a transcellular, route for UPEC passing the BTB and BEB.

### UPEC Infection Impairs Testicular Functions

**Figure 3:**
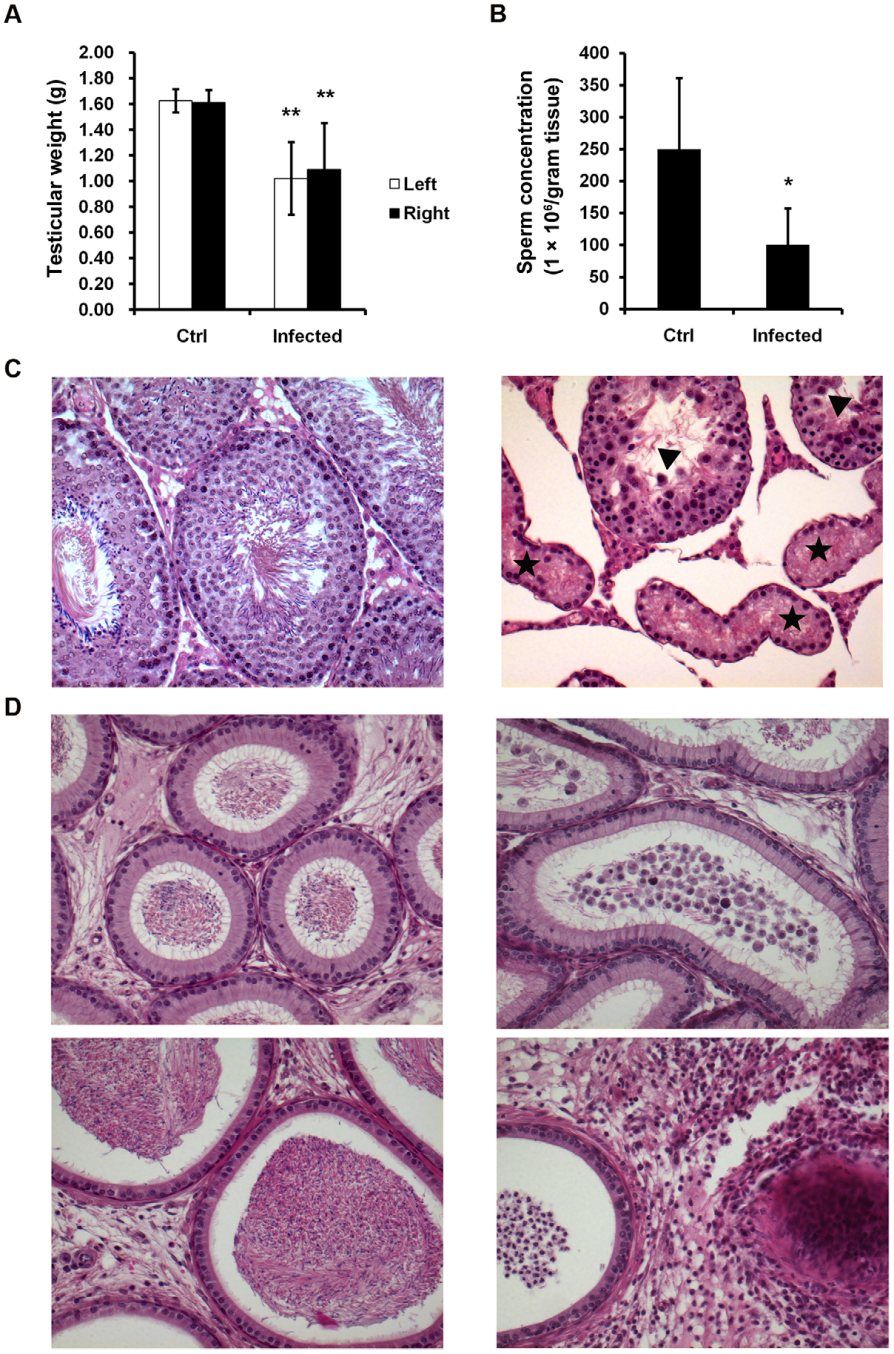
Morphological changes and histological evaluation of the testis and epididymis. (**A**) Testicular weight of control (n = 8) and infected rats (n = 10) are presented as mean ± standard deviation (SD). Student’s t-test was employed for statistical analysis and the level of significance is indicated as **p<0.001. (**B**) Sperm concentration was assessed in seven animals of each group and the results are presented as mean ± SD. Statistical analysis was performed with Student’s t-test and statistical significance is denoted as *p<0.05. (**C**) Tissue sections of paraffin embedded testes were stained with hematoxylin and eosin. Histopathological assessment was performed on control (n = 5) and UPEC infected (n = 9) testes using light microscopy. The images were captured using Axioplan 2 Imaging system at magnification x20 and representative figures are shown. Various forms of impairment of spermatogenesis are visible exemplified by a Sertoli cell only tubule (star) and a hypospermatogenic tubule (triangle). (**D**) Histopathological images of caput (D top panels) and cauda epididymis (D bottom panels, x20 objective). Representative results from control (n = 5) and infected (n = 9) rats are depicted.

Seven days following treatment, epididymides (swollen cauda) and testes (atrophy, increased vascularization with dilated vessels as well as vasocongestion) as clear macroscopical indications of infection were observed in UPEC injected rats (Figure S1A,B). Accordingly, the weight of both testes was found to be decreased by about 30% in UPEC infected rats compared to the saline injected sham control animals (left: 1.02±0.28 vs. 1.63±0.09 g; right: 1.09±0.36 vs. 1.61±0.10 g, *p*<0.001; Figure 3A). Furthermore, a clear reduction of the concentration of spermatozoa retrieved from the cauda epididymidis was observed at day 7 after infection (Figure 3B) compared to control rats indicating impairment of spermatogenesis (100.2±56.8×10^6^ vs. 249.8±111.6×10^6^/g tissue, *p*<0.05). To investigate the damage caused by UPEC infection, hematoxylin & eosin (H&E) stained sections from testes and epididymides were evaluated using light microscopy. Histopathological examination revealed gross morphological alterations such as edema formation and impairment of spermatogenesis to various degrees. Some tubules showed complete loss of germ cells (Sertoli cell only), whilst in neighboring tubules damage was milder with hypospermatogenesis and occurrence of multinucleated giant cells (Figure 3C). Detailed quantification demonstrated that in sections from infected testes 57.5% of tubules were damaged (in detail: 19.0% of tubules spermatogenesis proceeded up to mid elongated spermatid; in 10.1% of tubules germ cells developed until round spermatids; in 20.9% of seminiferous cord cross-sections spermatogenesis ceased at the stage of primary spermatocytes, whilst in 7.5% of tubules Sertoli cell only (SCO) or SCO with only a few remaining spermatogonia was evident, 42.5% of seminiferous tubules were intact (Figure 3C). Epididymal sections of the caput (Figure 3D, upper left panel) and cauda (Figure 3D, lower left panel) from saline injected rats show normal morphology of the epithelial layer and interstitial space. The lumen of the epididymides is filled with spermatozoa. In contrast, immature germ cells sloughed from infected testis appeared in the lumen of the caput/corpus epididymidis (Figure 3D, upper-right panel). Closer to the injection site in the cauda of the epididymis prominent signs of severe interstitial fibrosis, inflammatory cell infiltration and a high prevalence of immature germ cells with low numbers or even absence of spermatozoa is evident in infected rats (Figure 3D, lower-right panel).

### UPEC Infection Causes DNA Damage in Germ Cells

**Figure 4:**
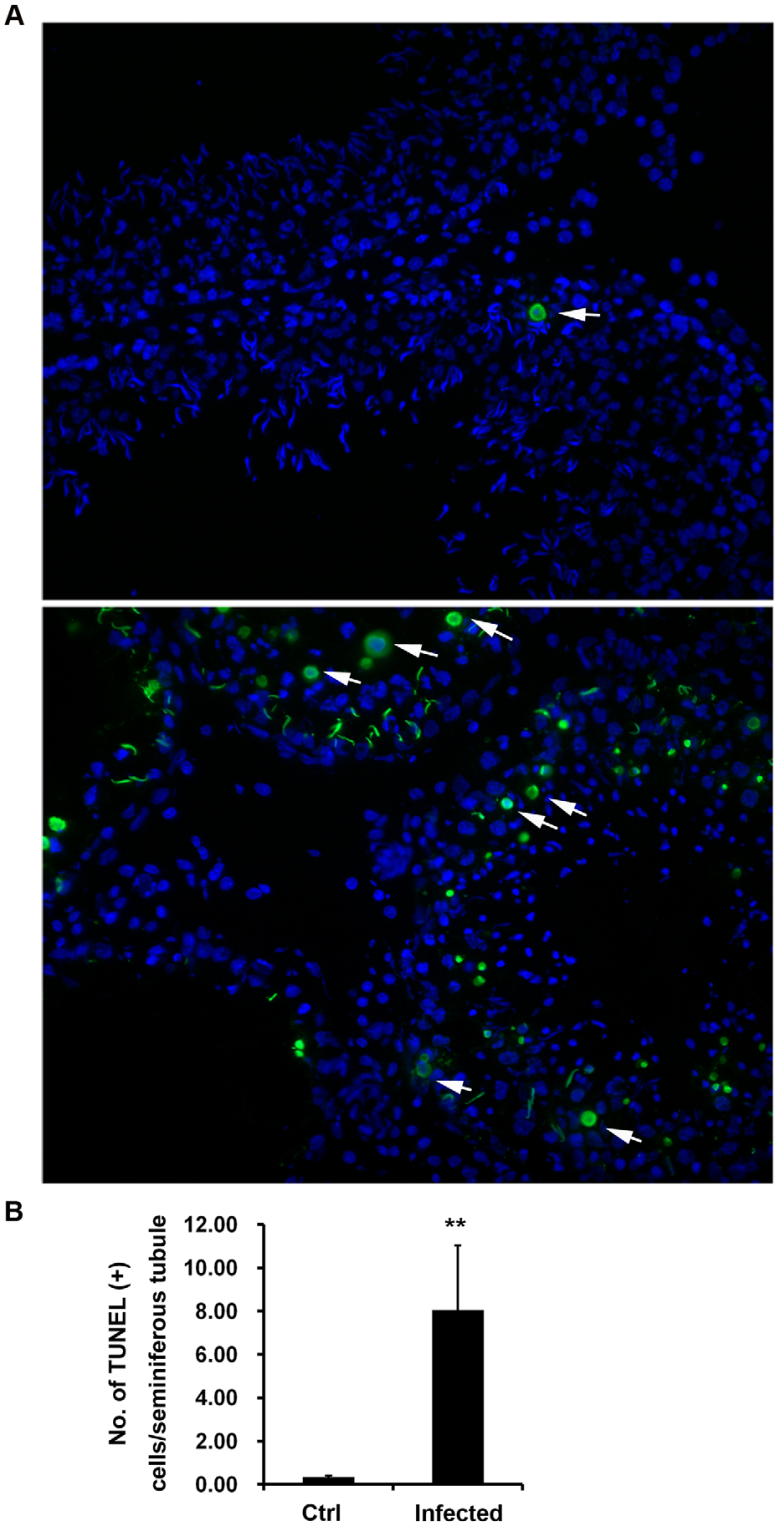
Increase of TUNEL positive cells in UPEC infected testis. (**A**) DNA strand breakage in testicular cells from control (upper panel) and UPEC infected (lower panel) rats were analyzed using TUNEL assay. Nuclei were counterstained with DAPI (blue). TUNEL (+) cells (green) with ring-like nuclear stain are indicated with arrows. (**B**) Numbers of TUNEL (+) cells are presented as mean ± SD/seminiferous tubule. Student’s t-test was used for statistical analysis and the level of significance is indicated as **p<0.01. (x20 objective).

Since apoptosis is the dominating mechanism in regulating germ cell death under normal conditions, it was initially explored whether apoptosis was implicated in UPEC induced impairment of spermatogenesis and germ cell loss *in vivo*. Therefore, TUNEL assay was carried out to detect DNA damage of testicular cells. The results (Figure 4A;B) indicate that the number of TUNEL positive cells increased more than 20 fold in infected testes compared to uninfected controls (8.05±2.99/tubule vs. 0.34±0.07/tubule, *p = *0.001). The majority of the TUNEL positive cells were located within the seminiferous epithelium with position and morphology typical for germ cells. Moreover, the typical ring type chromatin aggregation underneath the nuclear membrane as an indicator of early stage apoptosis [21] was also observed in some TUNEL positive cells (Figure 4A,B; arrows). Of note, there were a few TUNEL positive cells that lack the typical apoptotic ring-like nuclear structure suggesting that they were either at a different stage of apoptosis or alternatively undergoing necrosis.

### Expression Pattern of Apoptosis Related Genes in the Testis

**Figure 5:**
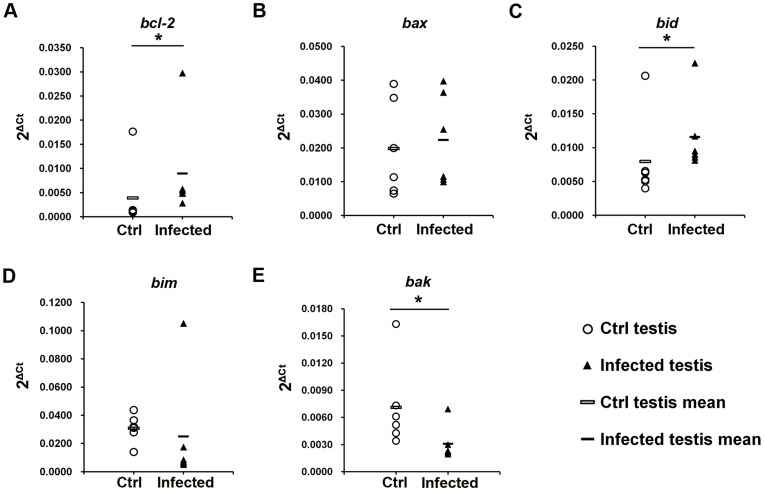
RNA expression pattern of the *bcl-2* family genes in the testis. The expression of the anti-apoptotic gene *bcl-2* (**A**), pro-apoptotic genes *bax* (**B**), *bid* (**C**), *bim* (**D**) and *bak* (**E**) in the testis were determined with quantitative real time PCR. Target gene expression levels were normalized with the endogenous control ß-2-microglobulin (ß2M). Data are present as 2^ΔCt^, ΔCt = Ct_target gene_-Ct_ß2M_. The Mann-Whitney U test was employed for statistical analysis (* *p*<0.05). Each single symbol (circle and triangle) represents one individual testis sample.

Based on the increase of DNA damaged cells seen by the TUNEL assay, it was next sought to delineate the specific mechanisms implicated in UPEC infection induced cell death in testis. *Bcl-2*, an anti-apoptotic gene, was upregulated about two times in the testis at day 7 post infection as determined by quantitative real-time PCR (relative expression in control v. s. infected testis: 0.0039 vs. 0.0090, *p = *0.037; Figure 5). From the proapoptotic genes, except for a slight upregulation of *bid* mRNA levels in infected testes (relative expression in control vs. infected testis: 0.0080 v. s. 0.0116, *p = *0.037, Figure 5C), no changes were observed in *bax* (relative expression in control vs. infected testis: 0.0198 vs. 0.0223, *p = *0.552, Figure 5B) and *bim* (relative expression in control vs. infected testis: 0.0309 vs. 0.0252, *p = *0.078, Figure 5D). The RNA level of another pro-apoptotic gene *bak* was even found to be slightly down regulated (relative expression in control vs. infected testis: 0.0071 vs. 0.0031, *p = *0.025; Figure 5E).

### Lack of Caspase Activation in Infected Testis

**Figure 6:**
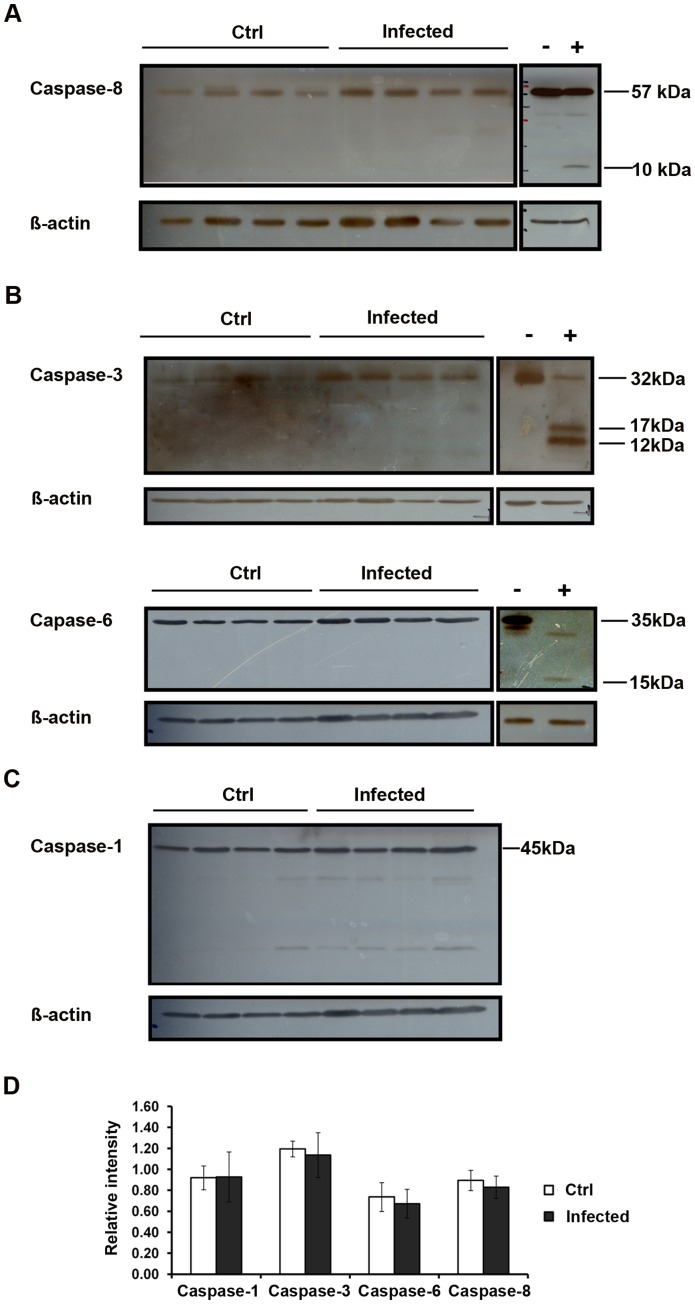
Caspase-1, -3, -6 and-8 are not activated in UPEC infected testis. Total testis protein (20 µg) from four different animals in each group were separated on 15% SDS-PAGE. Immunoblots were probed with anti-caspase-8 (**A**), anti-caspase-3 **(B, upper panel)**, anti-caspase-6 **(B, lower panel)** and anti-caspase-1 (**C**) antibodies and detected using chemiluminescence. RAW 264.7 cells treated with sodium nitroprusside (SNP) served as a positive control. (**D**) The intensity of target bands on the films was measured with the ImageJ software (http://rsbweb.nih.gov/ij/). Semi-quantitative results are presented as mean ± SD and Student’s t-test was used for data analysis (Caspase-8, *p = *0.875; Caspase-3, *p = *0.686; Caspase-6, *p = *0.486; Caspase-1, *p = *0.343).

Since caspase activation is a hallmark and essential step in apoptosis execution, the activation of these factors was assayed in total testis homogenates by Western blotting. As a positive control, RAW 264.7 cells were treated with 50 µM sodium nitroprusside (SNP) for 8 h to induce apoptosis. Activation of the extrinsic pathway is indicated by the cleavage of caspase-8. Surprisingly, the precursor protein of caspase-8 remained unchanged in all samples investigated and no caspase-8 cleavage products were detected, indicating that an activation of this initiator enzyme did not occur (Figure 6A,D). Furthermore, no activation of either the executor caspases-3 and -6, Figure 6B,D) nor of caspase-1 as key enzyme in the pyroptosis pathway (Figure 6C,D) were visible in infected testis.

### DNA Degradation in the Testis following UPEC Infection is Characteristic for Necrosis

**Figure 7:**
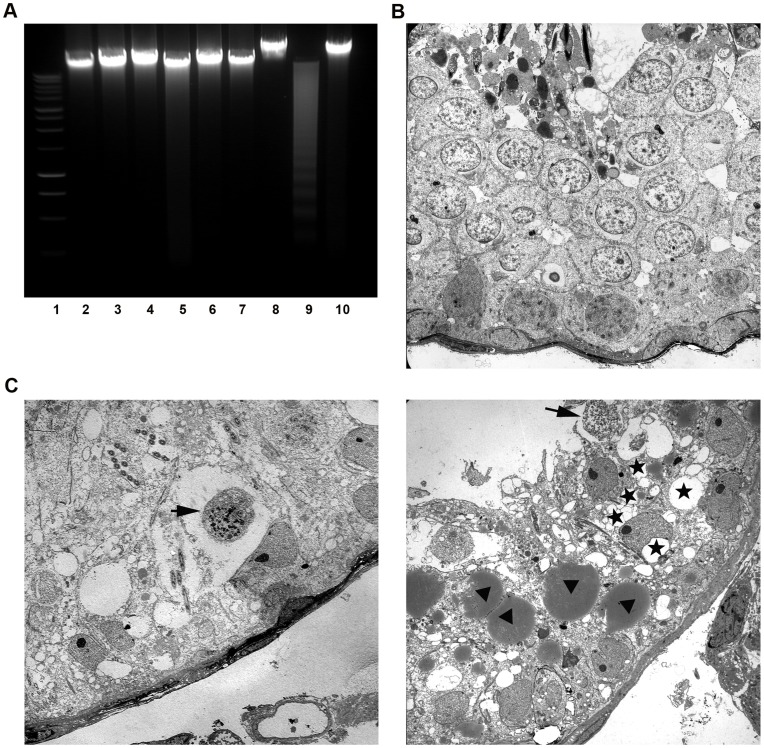
Oligonucleosomal DNA fragmentation measurement and ultrastructural examination of UPEC infected testis. (**A**) Genomic DNA was extracted from testes after seven days of infection. For gel electrophoretic analysis, 5 µg of DNA from each sample were separated on 1.5% agarose gels and stained with ethidium bromide. Lane 1∶1 kbp DNA ladder. Lane 2–4: DNA extracted from testes of control rats (n = 3). Lane 5–7: DNA samples extracted from testes of infected rats (n = 3). Lane 8: untreated RAW 264.7 cells. Lane 9: RAW 264.7 cells were treated with 0.5 mM H_2_O_2_ for 24 h as a positive control for apoptotic DNA laddering. Lane 10: RAW 264.7 cells were frozen and thawed repeatedly as a positive control for necrotic DNA fragmentation. (**B**) Electron microscopical examination of control rat testis shows normal morphology of the seminiferous epithelium (x1,100). (**C**) A representative ultrastructural image (x1,100) of infected testes demonstrates a hypospermatogenic seminiferous epithelium with germ cells displaying necrotic nuclei (arrows) and SC with strong cytoplasmic vacuolization (right panel, asterisk) and various large lipid droplets (right panel, arrowheads).

Genomic DNA degradation represents a late event in cell death with each death mode displaying a characteristic pattern. As an example, an orderly DNA fragmentation pattern is usually associated with caspase-dependent apoptosis. In apoptotic cells, the activation of endonucleases results in oligonucleosomal DNA fragments (DNA ladder) with steps of about 180 base pair (bp), while DNA is cleaved into fragments of random size by nonspecific lysosomal nucleases in necrotic cells. To determine which cell death mode plays the predominant role in UPEC infected testis, genomic DNA was analyzed by agarose gel electrophoresis. DNA laddering with approximately 180 bp fragmentation was visible only in RAW 264.7 cells treated with 5 µM H_2_O_2_ for 24 h (Figure 7A, lane 9), which served as a positive control for apoptotic DNA laddering. In saline injected control rats, total testis DNA remained intact (Figure 7A, lane 2–4), whereas the DNA samples extracted from infected testes (Figure 7A, lane 5∼7) did not show DNA laddering comparable to the apoptosis positive control (Figure 7A, lane 9).

### Electron Microscopical Examination Shows Typical Signs of Necrosis in Infected Testis

Whilst typical necrotic changes such as condensation of chromatin into small, irregular patches in poorly defined nuclei prevailed, indications for apoptosis were not seen in infected testis (Figure 7C). Of note, severe damage in SC was a frequent observation with vacuolization and accumulation of lipid droplets within the cytoplasm of SC as the most prominent signs (Figure 7D), which were not seen in control specimens (Figure 7B).

### HMGB1 is Released from the Nuclei in UPEC Infected Testis

**Figure 8:**
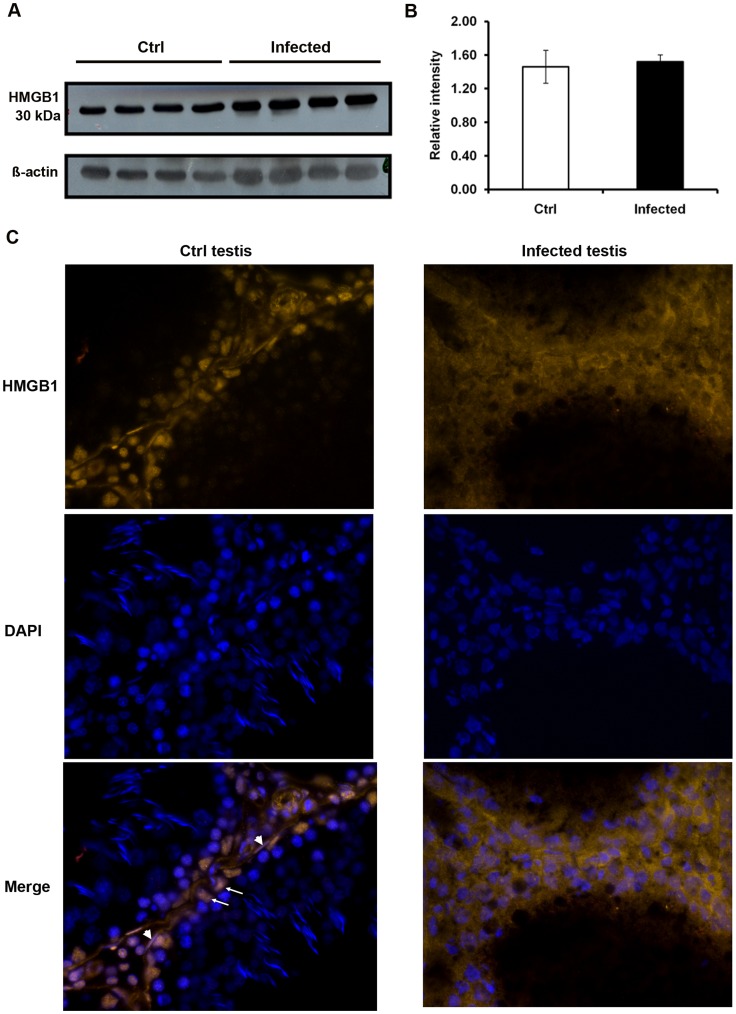
HMGB1 expression and localization in the testis. (**A**) For Western blot analysis, 20 µg of protein extracted from total testis of control (n = 4) and UPEC infected (n = 4) rats were separated on a 12.5% SDS-PAGE. HMGB1 was detected by immunoblot using anti-HMGB1 polyclonal antibody and chemiluminescence. ß-actin served as a loading control. (**B**) Intensity of target bands was measured with the ImageJ software (http://rsbweb.nih.gov/ij/) and data are presented as the relative intensity = intensity of HMGB1/intensity of ß-actin. (**C**) Testis cryosections were probed with anti-HMGB1 antibody decorated with Cy3-labeled secondary antibody (orange) and nuclei were counterstained with DAPI (blue, images taken with x40 objective). In control samples (left column) some Sertoli and peritubular cells are indicated by arrows and arrowheads, respectively. Representative results from two independent experiments are shown.

High-mobility group box 1 protein (HMGB1) has been identified as a marker of necrosis as it is passively released into the cytoplasm of necrotic or damaged cell, whilst it retains a nuclear localization in healthy and apoptotic cell [19]. Although the protein level of HMGB1 in total testis was not affected by UPEC infection (Figure 8A,B), different patterns of HMGB1 subcellular localization were observed between infected and control testes. In control testes, HMGB1 was exclusively found in the nuclei of somatic cells, i.e., SC (arrows), PTC (arrow heads), germ cells likely to represent spermatogonia and some interstitial cells (Figure 8C, left column) confirming previous data of Zetterström et al. [22]. In contrast, cytoplasmic and extracellular distribution of HMGB1 was observed in somatic cells of infected testes (Figure 8C, right column), thus indicating necrosis in the infected cells.

### NF-κB Signaling Pathway is not Involved in HMGB1 Release in UPEC Infected Testis

**Figure 9:**
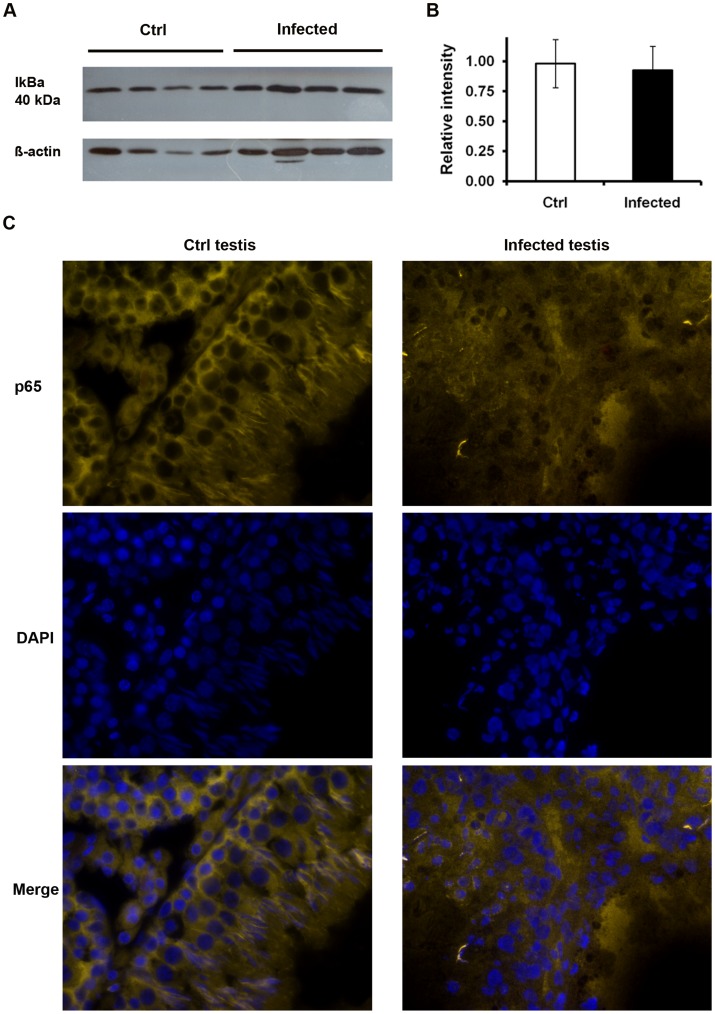
NF-κB pathway is not activated in UPEC infected testis. (**A**) Total testis proteins were separated on 10% SDS-PAGE. Immunoblots were labeled with mouse anti-IkBα antibody. ß-actin served as a loading control. (**B**) The intensity of target bands was measured with the ImageJ software (http://rsbweb.nih.gov/ij/) and results are presented as the relative intensity = intensity of p65/intensity of ß-actin. (**C**) Testis cryosections were probed with anti-p65 antibody labeled with Cy3-linked secondary antibody (orange) and the nuclei were counterstained with DAPI (blue, images taken with x40 objective). Representative results from at least two independent experiments are shown.

Recent work shows that activation of the proinflammatory NF-κB pathway is leading to regulated secretion of HMGB1 from the nuclei of intact activated macrophages as part of the inflammatory response [23]. Activation and nuclear translocation of NF-κB is essentially initiated by the degradation of its inhibitor IkBα. Seven days following UPEC infection, the protein levels of IkBα were comparable between UPEC infected and control testes (Figure 9A, B). In agreement, the p65 subunit of NF-κB was retained in the cytoplasm in all samples (Figure 9C). Similar observations were found in rats after one day and three days of infection (data not shown), indicating that the NF-κB pathway is not implicated in the previously observed release of HMGB1 and following cell death in this model.

## Discussion

Infection and inflammation of the male urogenital tract is established as an important etiological factor of infertility [13], [24]. Bacterial infections of the upper reproductive tract usually manifest as epididymitis or combined epididymo-orchitis [13], [25]. Relevant bacteria include *E. coli* pathovars usually also found in urinary tract infections and bacterial prostatitis such as alpha-hemolysin (HlyA) producing uropathogenic *E. coli* (UPEC). A recent survey revealed 40–50% of HlyA positive *E. coli* in the urine of epididymitis patients [3]. Affected men are often characterized by reduced numbers of germ cells and impairment of sperm functional parameters, which in a substantial proportion of patients persists even after successful clearance of the pathogen [26]. Nevertheless, the underlying mechanisms by which pathogens elicit damage are poorly understood. Using an established rat model of epididymo-orchitis, testes of animals infected with UPEC strain CFT073 at 7 days post infection demonstrated clear signs of inflammation manifested by swollen caudae epididymidis, atrophic testes with increased vascularization and vasocongestion. Degenerative changes were indicated by a reduced testicular weight in infected rats. Impairment of spermatogenesis was confirmed by a more than 50% reduction of spermatozoa numbers that could be retrieved from the cauda epididymis. Loss of capacity of the seminiferous epithelium to produce germ cells in UPEC infected rats was also indicated by the presence of numerous immature germ cells visible in the lumens of caput epididymidis. Presence of bacteria was identified by plating organ homogenates on LB-agar plates, by a PCR amplification of the PapC gene of UPEC and ultrastructurally. Surprisingly, in spite of the luminal inoculation of UPEC in the vas and the occurrence of bacteria in the lumen of the epididymis one day after infection (data not shown), at 7 days post infection the vast majority of UPEC was localized in the testicular interstitial space and not within the lumen or wall of the seminiferous epithelium. Based on a study of Chassin *et al.* [27], who demonstrated that UPEC CFT073 can pass the epithelial barriers of murine renal medullar collecting duct cells by disruption of the tight junctions, we investigated the integrity of the blood testis barrier (BTB) and the blood epididymis barrier (BEB) using lanthanum tracer injection. At least for this time point, we could not detect any leakage of tracer indicating that probably other mechanisms may be responsible for the passage of UPEC from the lumen and subsequent dissemination in the interstitial space. This could involve epithelial transcytosis which has been shown for other *E. coli* strains such as HT91 and HT7 [27]. An alternative option is a transient disruption of the BTB followed by restructuring before day 7 post infection, an event that occurs in a regulated manner during the transit of early meiotic cells through the barrier on their way from the basal to the adluminal part of the seminiferous epithelium. This process was found to be regulated by transiently upregulated TNF-α secreted from SC and germ cells [28]. In support, levels of TNF-α mRNA were significantly elevated in UPEC infected testis (Figure S2D). An explanation why UPEC are almost exclusively found in the interstitial space 7 days post infection could be derived from the expression pattern of anti-bacterial defensins which could generate a spatial selection pressure. Whilst all defensins found in the testis are expressed in the seminiferous epithelium, partly at high levels, defensin expression in the interstitial space is fairly faint [29].

To understand the molecular mechanisms of damage caused by UPEC infection, TUNEL assay was employed. A dramatic increase of TUNEL (+) testicular cells was seen in infected rats with the majority of TUNEL (+) cells located in the seminiferous epithelium. Based on their localization in the germinal epithelium most TUNEL (+) cells appeared to be germ cells. TUNEL detects DNA strand breaks which could occur as a late event in apoptosis, but also in necrosis [21], [30]. In apoptotic cells the activation of endonucleases results in oligonucleosomal DNA fragments (DNA ladder) with graded steps comprising about 180 base pairs, while DNA is cleaved into fragments of random size by nonspecific lysosomal nucleases in necrotic cells. In our study, we could not detect the 180 bp laddering in DNA of infected cell indicating that apoptosis is not the main death pathway. Apart from necrotic germ cells, electron microscopical analysis revealed severe degeneration of SC in infected testis as visualized by cytoplasmic vacuolization and extensive lipid droplets accumulation. Vacuolization of SC is not uncommon in degenerating SC and reported in other models using toxins to occur from swelling and coalescence of intracellular membrane bound organelles, particularly the endoplasmic reticulum and vesicles [31], [32]. In contrast, the presence of numerous large lipid droplets in SC may be related to an increased phagocytic activity removing germ cell debris following germ cell damage upon UPEC infection, but could also indicate a perturbance of lipid metabolism [33].

Although it is increasingly acknowledged that necrosis and apoptosis demonstrate more morphological and biochemical similarities than initially thought, a distinction between the different modes of cell death is still very relevant. This is particularly evident by the fact that in contrast to apoptosis, necrosis triggers a sterile inflammation that could sustain an extended inflammatory response even after clearance of the pathogen. One of the molecules responsible for late stimulatory effects in the inflammatory cascade is HMGB1. This cytokine is a member of the damage-associated molecular pattern (DAMP) molecules released from necrotic cells [34]. Previous investigations reported that HMGB1 can be upregulated by LPS as a general inflammatory stimulus [35]. In our study the expression levels of HMGB1 in testicular homogenates remained unchanged when comparing infected and non-infected tissues, however, a clear cut shift from a nuclear to a cytoplasmic or extracellular localization of HMGB1 was visible in UPEC infected testis *in vivo*. Cytoplasmic translocation and secretion of HMGB1 involves NFκB pathway triggering [36], a mechanism that could be excluded in our study as neither a degradation of the NFκB inhibiting molecule IkBα nor a nuclear translocation of the p65 subunit of NFκB was observed in infected testis after 7 days post infection. These results suggest induction of a necrotic pathway in testicular cells, as apoptotic cells firmly retain HMGB1 within nuclei even after undergoing secondary necrosis and subsequent autolysis [19]. Extracellularly, HMGB1 becomes a pro-inflammatory molecule with high affinity to several receptors such as RAGE and TLRs [37], [38]. Therefore, released HMGB1 may serve as an inflammatory signal from necrotic testicular cells to neighboring cells sustaining the inflammatory response with associated subsequent further damage to fertility.

Taken together, our study indicates that after ascending to the testis UPEC causes necrosis as the dominant mechanism of cell death in the rat testis. This is indicated by a lack of caspase activation and oligonucleosomal DNA laddering, unchanged expression levels of pro-apoptotic genes, and ultrastructural damage characteristic of necrosis, as well as release of HMGB1 from nuclei of Sertoli cells *in vivo*. Our data indicate that inhibition of HMGB1 may be useful as a further treatment option following antibiotic therapy to limit the negative impact of microbial infection of the testis.

## Supporting Information

Figure S1 **Morphological changes in the rat epididymo-orchitis infection model.** Typical appearance of epididymides and testes from saline injected (left panel) and UPEC infected rats (right panel) are visible.
(TIF)

Figure S2 **Upregulation of pro-inflammatory cytokine expression levels in infected testes.** The expression pattern of cytokines IL-1α (A), IL-1ß (B), IL-6 (C) and TNF-α (D) in the testis were determined with quantitative real time PCR. Target gene expression levels were normalized with the endogenous control ß-2-microglobulin (ß2M). Data are present as 2^ΔCt^, ΔCt = Ct_target gene_-Ct_ß2M_. The Mann-Whitney U test was employed for statistical analysis (* *p*<0.05, **p<0.01). Each single symbol (circle and triangle) represents one individual testis sample.
(TIF)
