# Supplementary figures and images for: Necrosis Is the Dominant Cell Death Pathway in Uropathogenic Escherichia coli Elicited Epididymo-Orchitis and Is Responsible for Damage of Rat Testis
Source: PLoS One. 2013 Jan 2;8(1):e52919. doi: 10.1371/journal.pone.0052919 (PMC3534655; doi:10.1371/journal.pone.0052919)

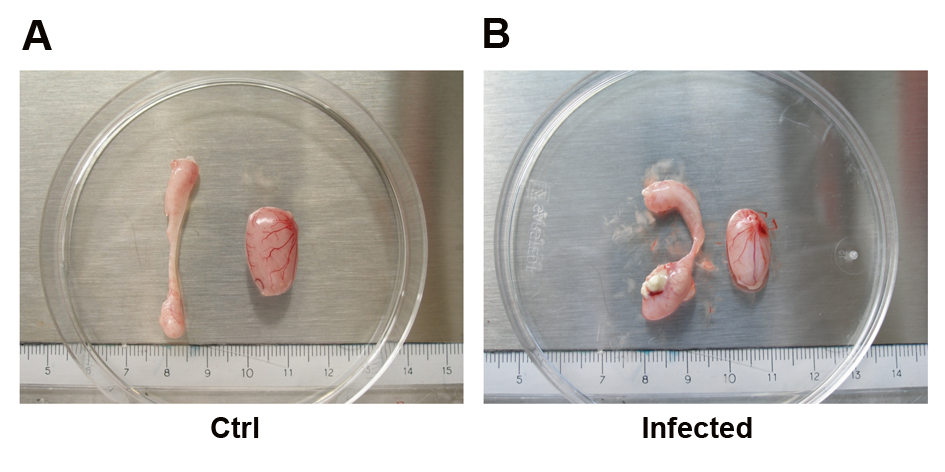

Supplement: Figure S1 — Morphological changes in the rat epididymo-orchitis infection model. Typical appearance of epididymides and testes from saline injected (left panel) and UPEC infected rats (right panel) are visible. (TIF) [file pone.0052919.s001.tif]

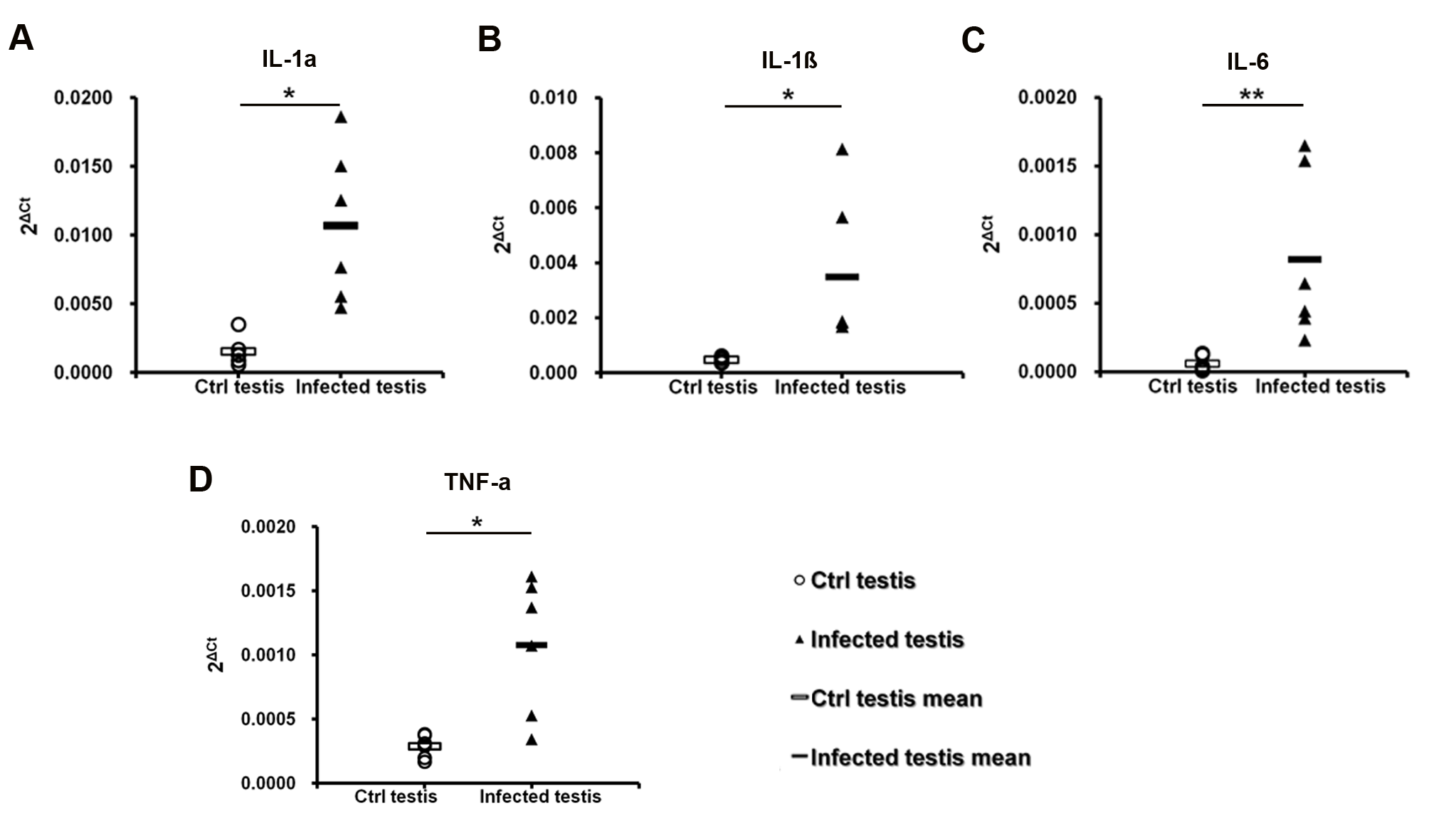

Supplement: Figure S2 — Upregulation of pro-inflammatory cytokine expression levels in infected testes. The expression pattern of cytokines IL-1α (A), IL-1ß (B), IL-6 (C) and TNF-α (D) in the testis were determined with quantitative real time PCR. Target gene expression levels were normalized with the endogenous control ß-2-microglobulin (ß2M). Data are present as 2ΔCt, ΔCt = Cttarget gene-Ctß2M. The Mann-Whitney U test was employed for statistical analysis (* p<0.05, **p<0.01). Each single symbol (circle and triangle) represents one individual testis sample. (TIF) [file pone.0052919.s002.tif]
